# Systematic review of health-related quality of life models

**DOI:** 10.1186/1477-7525-10-134

**Published:** 2012-11-16

**Authors:** Tamilyn Bakas, Susan M McLennon, Janet S Carpenter, Janice M Buelow, Julie L Otte, Kathleen M Hanna, Marsha L Ellett, Kimberly A Hadler, Janet L Welch

**Affiliations:** 1 Indiana University School of Nursing, 1111 Middle Drive, Indianapolis, IN, 46202, USA

**Keywords:** Health-related quality of life, Conceptual models, Theories, Frameworks

## Abstract

**Background:**

A systematic literature review was conducted to (a) identify the most frequently used health-related quality of life (HRQOL) models and (b) critique those models.

**Methods:**

Online search engines were queried using pre-determined inclusion and exclusion criteria. We reviewed titles, abstracts, and then full-text articles for their relevance to this review. Then the most commonly used models were identified, reviewed in tables, and critiqued using published criteria.

**Results:**

Of 1,602 titles identified, 100 articles from 21 countries met the inclusion criteria. The most frequently used HRQOL models were: Wilson and Cleary (16%), Ferrans and colleagues (4%), or World Health Organization (WHO) (5%). Ferrans and colleagues’ model was a revision of Wilson and Cleary’s model and appeared to have the greatest potential to guide future HRQOL research and practice.

**Conclusions:**

Recommendations are for researchers to use one of the three common HRQOL models unless there are compelling and clearly delineated reasons for creating new models. Disease-specific models can be derived from one of the three commonly used HRQOL models. We recommend Ferrans and colleagues’ model because they added individual and environmental characteristics to the popular Wilson and Cleary model to better explain HRQOL. Using a common HRQOL model across studies will promote a coherent body of evidence that will more quickly advance the science in the area of HRQOL.

## Introduction

Health-related quality of life (HRQOL) has been identified as a goal for all people across all life stages by leading health organizations [1-3]. HRQOL, that is, quality of life relative to one’s health or disease status, is a concern of policymakers, researchers, and health care practitioners [4]. Especially important is the need to align HRQOL research priorities with the needs and values of patients and their families. Because of the multidimensional aspects of HRQOL, and the varied use of this term across many different health and disease conditions, researchers have used a variety of HRQOL models to guide their research. A conceptual model is a schematic representation of a theory that acts as a heuristic device to provide a better understanding of a phenomenon (e.g., HRQOL) by depicting interrelationships among concepts [5]. The term conceptual model has been used interchangeably as “conceptual framework, theoretical model, or theoretically based conceptual model [6].”

There are many HRQOL models applied across different health and illness conditions, across the lifespan, and among individuals, their families, and communities. HRQOL is commonly conceived as dynamic, subjective, and multidimensional, and the dimensions often include physical, social, psychological, and spiritual factors [7]. However, the specific dimensions are labeled differently by different authors [7]. For example, these broad dimensions subsume more specific dimensions such as emotions, cognitive function, economic status, and intelligence [8], and they may incorporate friends and family [9]. While the theoretical underpinnings of HRQOL may be consistent across models, variations in terminology for analogous concepts make comparison across studies very difficult. Differing conceptualizations of HRQOL limit the ability to have a coherent body of evidence to guide further HRQOL research and practice. Common HRQOL models provide essential structure to the conceptualization of HRQOL using common language that can be shared across studies. Identification and evaluation of common HRQOL models can help guide research and practice toward promoting or attaining optimum HRQOL for populations of interest. Thus, the purposes of this paper were to (a) identify the most frequently used HRQOL models found in the literature over the past ten years and (b) review and critique the most commonly used models using established criteria by Bredow [10]. Although Bredow’s [10] criteria were developed to critique middle-range theories in nursing research, they represent a comprehensive approach to theory analysis for review and critique of HRQOL models [10]. The overall goal was to determine the relevance of HRQOL models to research and practice aimed at improving HRQOL.

## Methods

Several search engines were used to locate relevant articles. Initially, PubMed, MEDLINE, CINAHL, and PsychINFO were searched using the keywords quality of life, health-related quality of life, conceptual framework, conceptual model, and theory. Both quality of life and health-related quality of life were searched because these terms have been used interchangeably in the literature.

We limited our search to English language articles published between January 1, 1999 and August 31, 2010. The inclusion criteria were published articles pertaining to HRQOL models that had been used to guide (a) literature reviews, (b) instrument development studies, (c) descriptive or correlational studies, (d) intervention studies, or (e) practice. Articles in which research findings were used to derive HRQOL models were also included. We did not limit our search to specific populations (e.g., children, adolescents, adults, older adults) because we wanted a broad representation of the use of HRQOL models. Exclusion criteria were articles that did not pertain to humans, were non-English, or involved studies and information published only as dissertations, abstracts, editorials, or clinical opinion. Relevant articles were identified from the literature search using a three-step process. In the first step, authors working in pairs reviewed the article titles based on inclusion/exclusion criteria. In cases in which there was a lack of consensus between the two reviewers, a third reviewer was sought, consistent with methods outlined by the Joanna Briggs Institute [11]. In the second step, titles that met the criteria were further evaluated. Authors, again working in pairs, reviewed abstracts and reached agreement about whether the abstracts met inclusion/exclusion criteria. In the third step, the identified articles were obtained and evaluated by the same pairs of authors. Full text articles were reviewed and again were included only if the pairs agreed the article met the criteria.

The paired authors then extracted and consolidated pertinent information from the articles into a review table. Column variables in the table were: author and date, country of origin, purpose, methods, design, and model. Each row represented a unique article. All authors engaged in group discussion to reach consensus on articles to be included in the review and to determine the format in which to present review findings. After reviewing articles, the most commonly used HRQOL models were identified, fulfilling the first purpose of this review. For purpose two, each of the most commonly used HRQOL models was critiqued by the author pairs using established criteria by Bredow [10]. After considering several alternatives, we chose Bredow because he incorporated the most comprehensive criteria for evaluating theories, frameworks, and models [10]. Although these criteria are used to evaluate middle range theories in nursing research, they are also useful in evaluating quality of life theories [10]. A description of Bredow’s [10] criteria appears in the first column of Table 1, and is summarized below.

**Table 1:** Critique of three most commonly used HRQOL models

**Criteria and Description (Bredow, **[10]**)**	**Wilson &; Cleary Model of HRQOL**[12]	**Ferrans et al. Revised Wilson and Cleary Model of HRQOL**[13]	**World Health Organization International Classification of Functioning Disability and Health (WHO ICF)**[3]
**Internal criticism**			
**Adequacy**			
Addresses a defined area			
· Completeness	· Complete overall conceptualization of HRQOL from biomedical and social science perspectives	· Expanded Wilson &; Cleary’s model to better explicate individual and environmental factors	· Complete overall conceptualization of health from biomedical and social science perspectives
· Gaps	· Gaps include management of therapeutic regimens and self-management	· Gaps still include management of therapeutic regimens and self-management	· Gaps include determinants of health, management of risk factors, and self-management
· Need for refinement	· Refinement for specific practice situations needed.	· Refinement for specific practice situations needed.	· Refinement for specific practice situations needed.
**Clarity**	· Main concepts well-defined, although individual and environmental characteristics not explained.	· Main concepts well-defined, including individual and environmental characteristics.	· Main concepts well-defined, with the exception of overlap between activities and participation.
Explicit components	· Explicit proposition that dominant relationships exist with the potential for reciprocal relationships.	· Explicit proposition that dominant relationships exist with the potential for reciprocal relationships.	· Explicit propositions exist with reciprocal relationships that can be used to map the constructs and domains.
· Concepts (components) defined	· Strength of the relationships of each component is unclear and with each additional relationship the complexity increases.	· Propositions were added with individual and environmental characteristics.	· Explicit assumption that model provides a multipurpose classification and can serve as a unified and standard language for health care workers, researchers, policy-makers, and the public.
· Explicit propositions (Relationships)	· Other relationships were implied.	· Nonmedical factors removed; described as part of individual and environmental characteristics.	
· Explicit assumptions (Beliefs)	· Explicit assumption that understanding relationships among these domains will lead to the design of optimally effective clinical interventions.	· Explicit assumption that understanding relationships among these domains will lead to the design of optimally effective clinical interventions.	· Another explicit assumption is that model can be used to help plan interventions for functional goals and health, worldwide.
**Consistency**			
Consistency			
· Concepts Congruency	· Concepts consistently defined.	· Concepts consistently defined.	· Concepts consistently defined.
· Assumptions (beliefs)	· Assumptions were congruent	· Assumptions were congruent	· Assumptions were congruent
· Propositions (relationships)	· The figure depicts dominant directional relationships whereas the text mentions reciprocal and other non-depicted relationships.	· Propositions were congruent.	· Propositions were congruent.
**Logical development**	· Emerged based on research from biomedical and social sciences.	· Revision of Wilson &; Cleary	· Integration of medical and social models for a biopsychosocial approach.
Based on previous work Evidence supports	· Relationships depicted don’t always hold true, research evidence supports lack of relationships in some instances (e.g., biological vs. symptoms)	· Emerged based on empirical evidence and the need for further clarity.	· Evolved over time from the WHO ICIDH model in 1980 to the WHO ICF in 2001, with the WHO ICF-CY for children and adolescents added in 2007.
			· Based on systematic field trials and international consultation.
**Level of development**			
Level of abstraction (grand, middle range, or practice)	· Middle range but global	Middle range but global	Middle range but global
**External criticism**			
**Complexity**			
· Number of concepts	· 5 main abstract concepts (biological/physiological, symptom status, functional status, general health, quality of life)	· 5 main abstract concepts with further development of the individual and environmental factors.	· 6 main abstract concepts (body functions, body structures, activity, participation, environmental factors, and personal factors).
· Parsimony	· Parsimonious because used only 5 main concepts to explain abstract HRQOL.	· Parsimonious because used only 7 main concepts to explain abstract HRQOL.	· Parsimonious because used only 6 main concepts to explain abstract health and health-related states.
· Complexity	· Overall model is complex with multiple relationships	· Overall model is complex with multiple relationships	· Overall model is complex with multiple relationships
**Discrimination**	· First HRQOL model to combine biomedical with social science	· Revised Wilson and Cleary’s HRQOL model	· Belongs to a family of WHO Classifications, with the WHO ICF being specific to functioning and disability.
Unique theory of HRQOL with clear boundaries	· Unique to HRQOL	· Unique to HRQOL	· Not unique to HRQOL.
	· Boundaries are purposefully not clear as two theories are combined and the relationships between concepts are additive.	· Clear boundaries and limited to HRQOL of individuals.	· Clear boundaries addressing health and health-related domains.
	· Hypotheses generation may help to clarify boundaries.		· Does not cover non-health related circumstances.
**Reality convergence**	· Moving from cellular level to quality of life in model seems more realistic than traditional biomedical model by itself.	· Realism added with the incorporation of nonmedical factors into individual and environmental factors.	· Assumptions seem true, realistic, and consistent.
· Assumptions “real world”	· “Makes sense” for real world application.	· “Makes sense” for real world application.	· “Makes sense” for real world application.
· Theory/model “makes sense”	· Assumptions are difficult to actualize	· Assumptions more realistic	
**Pragmatic**	Guided literature applied to real world settings:	Guided literature applied to real world settings:	Guided literature applied to real world settings:
Operationalized in real-life settings	· 3 literature reviews,	· 2 literature reviews	· 3 literature reviews
	· 4 descriptive,	· 1 instrument development	· 2 instrument development
		Model testing in entirety not done	Model testing in entirety not done
	· 6 correlational,	· Overall, generic and situation-specific measures exist	· Overall, generic and situation-specific measures exist
	· 1 randomized trial,	· Response shift is a concern for general health and quality of life components	· Response shift may also be a concern.
	· 1 qualitative,		
	· 1 mixed methods		
	· 1 model revision (Ferrans) Model testing in entirety rarely done		
	· Overall, generic and situation-specific measures exist		
	· Response shift is a concern for general health and quality of life components		
**Scope**			
· breadth of theory/model	· Broad model to explain complex nature of HRQOL	· Further broadens Wilson and Cleary’s scope by expanding on individual and environmental factors	· Broad model to explain health and health-related domains for all people.
· applies across ages (lifespan), health and disease conditions, cultures, socioeconomics, and individuals/families/ communities	· Could apply to individuals of all ages, life spans, health and disease conditions, and perhaps cultures depending on their orientation to the meaning of quality of life and general health.		· Could apply to individuals of all ages, life spans, health and disease conditions, and cultures across the world.
			· WHO ICF-CY specifically covers infants, children, and adolescentzs.
	· May not apply to those who are unable to define their own general health or quality of life (e.g., infants, comatose), or those who have very limited functioning.		· Focus is on individuals (with or without disabilities), families, communities, and populations.
	· Primarily applies to individuals, less to families and communities.		
**Significance**	· Most widely cited HRQOL model	· Emerging citations for Revised HRQOL model	· Emerging citations for the use of the WHO ICF for hypothesis testing (mainly instrument development).
· Potential impact on practice	· Guides HRQOL assessment toward a more comprehensive approach to improving HRQOL Potential for intervention research but limited evidence exists to date.	· Guides HRQOL assessment toward a more comprehensive approach to improving HRQOL	· As a clinical tool, can be used for needs assessments, matching treatments with conditions, and evaluating outcomes.
· Hypotheses lead to assessment or interventions	· Because of the complexity of the model and lack of testing of the full model, supporting interventions would be difficult.	· Potential for intervention research but limited evidence exists to date.	· As a research tool, can be used for measuring quality of life, outcomes, environmental factors, or other constructs.
			· Potential for intervention research but limited evidence exists to date. More of a mapping and classification framework, rather than hypothesis generating.
**Utility**	Hypothesis generating for:	Hypothesis generating for:	Hypothesis generating for:
Hypothesis generating for clinicians, researchers, epidemiologists, policymakers	· Clinicians for a broader view of HRQOL than just biological factors and symptoms.	· Clinicians for a broader view of HRQOL than just biological factors and symptoms.	· Clinicians for needs assessments, matching treatments with conditions, vocational assessment, and rehabilitation and outcome evaluation
	· Researchers to guide measurement and intervention studies:	· Expands focus of article (audience) from physicians (Wilson &; Cleary) to nurses and other health professionals (Ferrans). Model could be applied to any health care discipline.	· Researchers to guide development of measures for outcomes, quality of life, or environmental factors
	· Potentially relevant to epidemiologists if using global measures across populations (e.g., SF-36).	· Researchers to guide measurement and intervention studies.	· Epidemiologists to collect and record data for populations and management information systems
	· More research evidence and emphasis on environmental factors needed to convince policymakers.	· Potentially relevant to epidemiologists if using global measures across populations (e.g., SF-36).	· Policymakers to plan social security, compensation systems, and policies.
		· More research evidence and emphasis on environmental factors needed to convince policymakers.	· Educators to design curriculums that emphasize awareness and social action.
			· Although potential for hypothesis generation in these areas, there is currently limited evidence found in the HRQOL literature documenting these applications.

Bredow’s [10] criteria for evaluating theories were organized around two major areas: internal and external criticism. Internal criticism involves a judgment about the internal components of the theory, whereas external criticism involves a judgment about the match between the theory and context of its use. When evaluating internal criticism, the evaluator assesses the adequacy (thoroughness in addressing topic), clarity (clearness of statements), consistency (congruency in semantics, etc.), logical development (support from evidence), and level of theory development. To make judgments about external criticism, the evaluator assesses the complexity (number of concepts/variables, from parsimonious to complex), discrimination (uniqueness), reality convergence (relevant assumptions), pragmatism (ability to use in the real world), scope (narrow to broad use for practice), significance (impact of theory), and utility (ability to produce hypotheses). Critique information for each of the commonly used models was summarized in a table after consensus had been reached by two (or sometimes three) authors.

## Results

**Figure 1:**
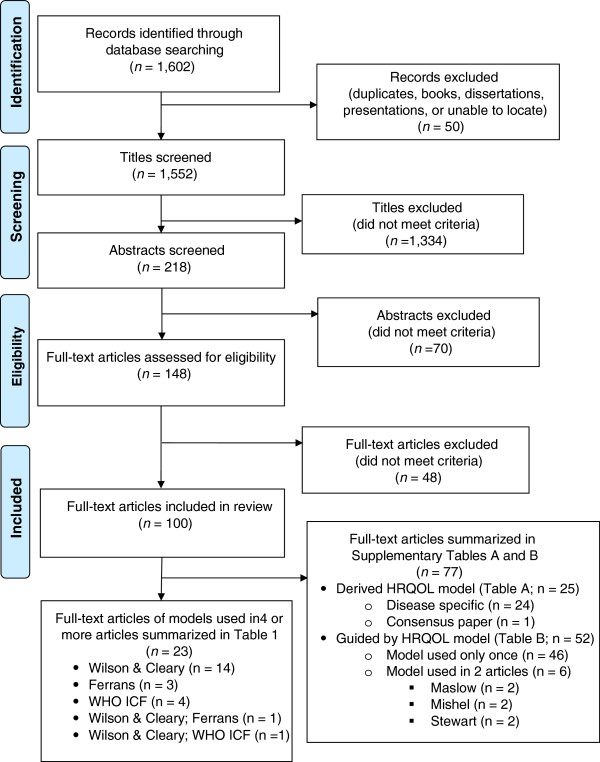
Literature search flow diagram.

The disposition of the search results is shown in Figure 1 as a PRISMA flow diagram [14]. Searching the three databases with the selected keywords yielded a total of 1,602 records. Author review excluded 50 records because they were duplicates, books, dissertations, presentations, or could not be located. This left 1,552 titles to screen. Author pairs excluded 1,334 titles because they did not meet inclusion criteria. This left 218 abstracts to be screened, of which 70 did not meet inclusion criteria; 148 progressed to the full text assessment for eligibility. Of the 148 full text articles assessed, 48 were eliminated because a HRQOL model had not been derived from or used to guide the research, review, and/or findings. This process resulted in a total of 100 articles being included in this review (see Figure 1).

Of the 100 articles, 46 were quantitative. Of the remaining 54, 16 were qualitative research, 1 was mixed methods research, 15 involved instrument development, 20 were literature reviews, 1 described a model revision, and 1 was a consensus paper. The 46 quantitative studies were mainly descriptive studies (*n* = 31), with a few being correlational (*n* = 13), or randomized controlled trials (*n* = 2). Sample sizes ranged from 10 [15] to 69,031 participants [16]. The 100 articles came from 21 different countries including Australia (*n* = 4), Austria (*n* = 1), Brazil (*n* = 3), Canada (*n* = 12), China (*n* = 3), Finland (*n* = 1), Germany (*n* = 4), India (*n* = 1), Ireland (*n* = 1), Israel (*n* = 1), Italy (*n* = 2), Japan (*n* = 1), Netherlands (*n* = 7), Norway (*n* = 2), Spain (*n* = 3), Sweden (*n* = 2), Taiwan (*n* = 1), Thailand (*n* = 4), Ukraine (*n* = 1), United Kingdom (*n* = 10), and the United States (*n* = 49). Of these 100 articles, 9 involved more than one country.

### Most frequently used HRQOL models

Of the 100 articles, 57 used an existing HRQOL model as a guide and 25 derived a HRQOL model. Interestingly, 18 articles used an existing HRQOL model as an initial guide and then also derived a revised model based on the findings. Figure 1 shows that of the 100 full-text articles included in the review, 77 either derived a HRQOL model (n = 25) or were guided by a HRQOL model that was used only once or twice (n = 52). There was little consensus among the models used, with each article essentially citing a different model. These 77 articles are summarized in Additional file 1: Tables SA and SB included as an appendix for this paper. Of the 25 articles that derived a HRQOL model, 24 were disease-specific, and 1 was a consensus paper on HRQOL (Additional file 1: Table SA). The disease-specific models were classified as using a uniquely derived HRQOL model based on the findings. For example, Barr and Schumacher [17] identified six categories of HRQOL specific to individuals receiving medical nutrition therapy. Similarly, Klassen, Pusic, Scott, Klok, &; Cano [18] examined the impact of breast conditions and surgery to develop a quality of life framework specific to breast surgery patients. Because there was such a wide variation in disease states, HRQOL domains, and particular characteristics, findings could not be adequately synthesized. Of the 52 articles that were guided by a HRQOL model that was used only once (n = 46) or twice (n = 6), only three HRQOL models were cited twice (Additional file 1: Table SB). Those used twice were Maslow’s hierarchy of needs (n = 2), Mishel’s Uncertainty in Illness Theory (n = 2), and Stewart’s conceptual model of factors affecting dying patients and families (n = 2) (See Additional file 1: Table SB for details).

As depicted at the bottom of Figure 1, there remained a total of 23 articles that cited the same model 4 or more times. As shown in Table 2 (and in Figure 1), the most common existing HRQOL models found in the literature were those by Wilson and Cleary [12] (*n* = 14), Ferrans and colleagues [13,19] (*n* = 3), and the World Health Organization (WHO) [20,21] (*n* = 4). Two additional articles used a combination of two of these models. Ferrans et al. [13] used the Wilson and Cleary [12] model as a guide to derive a revised model of HRQOL [13]. Valderas and Alonso [22] used both Wilson and Cleary [12] and the WHO [20] models. Schematic diagrams for each of the three most common HRQOL models have been published in Wilson and Cleary [12], Ferrans and colleagues [13], and the World Health Organization [20,21], and are described in more detail in the results section. The largest group (*n* = 10) of the 23 articles in Table 2 reported observational studies (descriptive or correlational) and focused on patients with chronic illness, with sample sizes ranging from 61 [23] to 917 [24]. Literature reviews (*n* = 6) and instrument development studies (*n* = 3) were also found. Only one randomized controlled trial was found [25], along with one mixed-methods study [26], one qualitative study [27], and one article that described a model revision [13].

**Table 2:** Subset of articles based on three most commonly used HRQOL models

**Model used [Reference]**	**Authors [Reference] Country**	**Purpose**	**Design**	**Sample**	**Model guided or derived**
Wilson &; Cleary [12]	Baker, Pankhurst, &; Robinson [25] United Kingdom	To test Wilson and Cleary’s conceptual model of the direct and mediated pathways between clinical and non-clinical variables in relation to oral health-related quality of life (OHRQOL).	Randomized Controlled Trial	85 patients with xerostomia attending outpatient clinics	Guided
Wilson &; Cleary [12]	Cosby, Holzemer, Henry, &; Portillo [28] United States	To determine relationships among anemia, neutropenia, and thrombocytopenia and the five dimensions of the Wilson and Cleary model of HRQOL.	Correlational	146 hospitalized patients with AIDS	Guided
Wilson &; Cleary [12]	Frank, Auslander, &; Weissgarten [29] Israel	To examine quality of life among patients undergoing different types of treatment for end-stage renal disease at different points of the disease.	Descriptive	72 patients with end-stage renal disease	Guided
Wilson &; Cleary [12]	Hofer, Benzer, Alber, Ruttmann, Kopp, Schussler, et al. [30] Austria, Ireland, Germany	To apply the Wilson and Cleary model a priori to patients with coronary artery disease.	Correlational	465 patients with coronary artery disease	Guided
Wilson &; Cleary [12]	Janz, Janevic, Dodge, Fingerlin, Schork, Mosca, et al., [31] United States	To describe the impact of clinical and psychosocial factors on the quality of life of older women with heart disease.	Descriptive	570 older women with heart disease	Guided
Wilson &; Cleary [12]	Krethong, Jirapaet, Jitpanya, &; Sloan [32] Thailand	To examine causal relationships among bio-physiological status, symptoms, functional status, general health perception, HRQOL, and social support.	Correlational	422 Thai patients with heart failure	Guided
Wilson &; Cleary [12]	Mathias, Gao, Miller, Cella, Snyder, Turner, et al. [27] United States	To develop a conceptual model to describe the impact of immune thrombocytopenic purpura on HRQOL.	Qualitative &; Literature Review	23 patients with immune thrombocytopenic purpura	Guided Derived
Wilson &; Cleary [12]	Mathisen, Andersen, Veenstra, Wahl, Hanestad, &; Fosse [33] Norway	To determine whether reciprocal relationships existed between quality of life and health appraisal in those with coronary artery bypass surgery.	Correlational	120 patients with coronary artery bypass surgery	Guided Derived
Wilson &; Cleary [12]	Orfila, Ferrer, Lamarca, Tebe, Domingo-Salvany, &; Alonso [34] Spain	To determine whether gender differences in HRQOL among the elderly are explained by differences in performance-based functional capacity and chronic conditions.	Descriptive	544 elderly persons	Guided Derived
Wilson &; Cleary [12]	Penckofer Ferrans, Fink, Barrett, &; Holm [23] United States	To determine effect of coronary artery bypass Graft (CABG) surgery on quality of life of women.	Descriptive	61 women with coronary artery bypass Surgery	Guided
Wilson &; Cleary [12]	Sousa [35] United States	To describe a HRQOL model to guide clinical practice.	Literature Review	NA	Guided
Wilson &; Cleary [12]	Sousa &; Kwok [24] United States	To validate Wilson &; Cleary’s model using structural equation modeling in HIV+ patients.	Correlational	917 HIV+ patients	Guided
Wilson &; Cleary [12]	Vidrine, Amick, Gritz, &; Arduino [36] Israel	To empirically assess a proximal-distal framework for conceptualizing HRQOL in individuals living with HIV/AIDS. An integrated model based on Wilson and Cleary (2005) was used.	Correlational	348 people with HIV/AIDS	Guided Derived
Wilson &; Cleary [12]	Wettergren, Bjorkholm, Axdorph, &; Langius-Eklof, [26] Sweden	To examine determinants of HRQOL in long-term survivors of Hodgkin’s Disease.	Mixed Methods	121 long-term Hodgkin’s lymphoma survivors + 236 healthy controls	Guided
Ferrans et al. [13]	Daggett, Bakas, &; Habermann [37] United States	To identify gaps in current knowledge of HRQOL and traumatic brain injury and apply findings to developing recommendations for future research with combat veterans with traumatic brain injury.	Literature Review	N/A	Guided
Ferrans &; Powers [19]	Hill, Aldag, Hekel, Riner, &; Bloomfield [38] United States	To develop and test psychometric properties of a maternal post-partum quality of life measure.	Instrument Development	184 post partum mothers	Guided
Ferrans &; Powers [19]	Petchprapai &; Winkelman [39] Thailand United States	To analyze the literature related to the clinical, theoretical, and empirical determinates of mild traumatic brain injury.	Literature Review	N/A	Guided
World Health Organization [20]	Fischer, LaRocca, Miller, Ritvo, Andrews, &; Paty [40] Canada &; United States	To (1) review recent efforts to assess the broader impact of MS on quality of life; (2) describe the development of the MS Quality of Life Inventory (MSQLI); (3) discuss issues to consider in selecting an MS quality of life instrument.	Instrument Development	15 MS patients in pilot test, 300 MS patients in field test	Guided
World Health Organization [21]	Hays, Hahn, &; Marshall [41] United States	To examine different conceptual models of HRQOL and examine implications of these perspectives for measurement of HRQOL in persons with disabilities.	Literature Review	N/A	Guided
World Health Organization [20]	John [15] Germany	To explore the dimensional structure of OHRQOL using experts’ opinions using a conceptual model of oral health.	Instrument Development	10 dentists &; 4 psychologists	Guided
World Health Organization [20]	Post, deWitte, &; Schrijvers [42] Netherlands	To extend the World Health Organization international classification of impairments, disabilities, and handicaps in rehabilitation.	Literature Review	N/A	Guided Derived
Wilson &; Cleary [12]; Ferrans et al., [13]	Ferrans, Zerwic, Wilbur, &; Larson [13] United States	To revise the Wilson and Cleary model of HRQOL.	Model Revision	N/A	Guided Derived
Wilson &; Cleary [12]; World Health Organization [20]	Valderas &; Alonso [22] United Kingdom Spain	To develop a classification system for patient-reported outcome measures based on Wilson &; Cleary’s HRQOL conceptual model and the World Health Organization International Classification of Functioning.	Literature Review	NA	Guided Derived

### Critical analysis of predominant HRQOL models

Table 1 details the critique of the three most commonly used HRQOL models found in the literature over the past 10 years using criteria by Bredow [10]. Wilson and Cleary’s [12] model of HRQOL combines two paradigms, biomedical and social science. Their model is a taxonomy that includes five major well-defined domains: biological, symptoms, function, general health perception, and overall HRQOL. However, the definitions for two other domains, individual and environmental characteristics, were not made explicit. Each domain is related to the others, and reciprocal relationships may exist. The authors suggest that environmental and individual factors are associated with outcomes, thus affecting total HRQOL.

Ferrans, Zerwic, Wilbur, and Larson [13] published a revision of Wilson and Cleary’s [12] HRQOL model. The five major domains of the original model were retained. Ferrans and colleagues [13] made explicit the definitions for individual and environmental characteristics, and they simplified the depiction of the model by removing non-medical factors and labels on the arrows portraying the relationships in the figure. In addition, they contributed further theoretical background about the main concepts in the model [19] and provided examples of instruments to enhance measurement. According to Ferrans et al. [13], the model depicts dominant causal associations; however, reciprocal relationships are implied. An explicit assumption is that understanding relationships among these components will lead to the design of optimally effective clinical interventions. The revised conceptual model could be applied to any health care discipline.

The World Health Organization International Classification of Functioning, Disability, and Health (WHO ICF) is a model designed to provide a description of health and health states, while providing a unified and standard language that can be used across disciplines and cultures [3,20,21]. The WHO ICF has evolved over time from a focus on “consequences of disease” in 1980 to “components of health” in 2001 [20,21]. The more recently developed WHO ICF-CY covers infants, children, and adolescents [3]. The WHO has conceptualized HRQOL as an individual’s perception of his or her health and health-related domains of well-being [3,21,43]. Health and health-related domains have been further conceptualized in terms of functioning within the WHO ICF model. The WHO ICF model includes components within two main parts. Part 1 focuses on functioning and disability (body functioning and structures, activities, and participation), whereas Part 2 addresses contextual factors (environmental and personal). The main concepts are well-defined overall, with explicit propositions and assumptions. However, unlike the models by Wilson and Cleary [12] and Ferrans and colleagues [13], the WHO ICF is not specific to HRQOL. Cieza and Stuki [43] assert that the WHO ICF categories under functioning can serve as the basis for the operationalization of HRQOL but are not the only potential application of the WHO ICF. For example, Miller and colleagues [44] used the WHO ICF as a framework to organize a comprehensive overview of nursing and interdisciplinary care of the stroke patient. The WHO ICF serves more as a mapping and classification framework than as a guide for hypothesis generation in the area of HRQOL.

Critique of the internal components of the HRQOL models using the Bredow criteria [10] indicated many similarities and some differences. All were fairly complete in the descriptions and definitions of HRQOL, with some gaps in the influence of management of therapeutic regimens and self-management on quality of life. Most existing models focus on the influence of symptoms rather than on management related to the condition. For example, for those with diabetes, both symptoms (such as hypoglycemia) and management (such as frequent checking of glucose levels) are important influential factors for HRQOL. Within the WHO ICF model [3], there was some definitional overlap between activities and participation. The Ferrans et al. [13] model was more complete and clear than that of Wilson and Cleary [12] because of the revisions and because of better definitions for individual and environmental factors. The relationships among the concepts were less clear in the Wilson and Cleary model [12], whereas the Ferrans et al. model [13] added clarity. Both the Wilson and Cleary [12] and Ferrans et al. [13] models implied potential reciprocity. In contrast, the WHO ICF [3] was explicit with the depiction of causal and reciprocal relationships.

There were greater variations among the three models when critiquing model fit with operational application. The models were similarly parsimonious, yet the complexities of multiple relationships were described. They made sense for use in real-world settings and have been used to guide research and practice. A major difference is that the Wilson and Cleary [12] and Ferrans et al. [13] models specifically explain HRQOL, whereas the WHO ICF model [3] describes health related to functioning and disability. In addition, though the Wilson and Cleary [12] and Ferrans et al. [13] models were primarily intended for application to individuals, the WHO ICF model [3] could be used to explain the health of individuals, families, communities, populations, and cultures. With the former, adaptations may be needed for use with families, communities, and individuals unable to report their own HRQOL, such as infants and young children and those with cognitive impairment. Empirical evidence for use of the models for intervention research is limited. However, the Ferrans et al. [13] model and the WHO ICF model [3] have robust potential for guiding the design of interventions that could be tested and applied in practice settings. The WHO ICF may be more applicable to practice situations for needs assessments, matching treatments with conditions, and evaluating outcomes because it is primarily a classification and mapping system. All three models were at a similar level of development emerging from the two paradigms of biomedical and social sciences.

## Discussion and recommendations

There are two important findings from this review. First, there has been little consistency in HRQOL models within the literature of the past 10 years. Approximately three-fourths of the articles reviewed used an existing HRQOL model as a guide; however, most of these applied a variety of different models, rather than using a common model found in the literature such as the Wilson and Cleary model [12]. Thus, there were wide variations in terminology for analogous HRQOL concepts, making cross-study comparisons virtually impossible. This seriously limits the ability to have a coherent body of evidence to guide further HRQOL research and practice. Second, the most commonly used models were based on work by Wilson and Cleary [12], the revised model by Ferrans and colleagues [13,19], and the WHO [3,20,21]. A majority of the researchers using these models could be doing so because of an absence of better alternatives. However, based on our findings, we recommend that authors consider the advantages of using one of the three commonly used global models in research to more quickly advance the science in the area of HRQOL. Our findings show that Wilson and Cleary [12], as well as the revisions of Wilson and Cleary’s model proposed by Ferrans et al. [13], together are the most frequently referenced in the HRQOL literature, representing nearly a quarter of all of the articles reviewed. Ferrans and colleagues’ [13] model provides clear conceptual and operational definitions, and it also clarifies relationships among concepts to guide research and practice. The WHO ICF model [3] may be useful in specific HRQOL studies; however, it has more potential for application to studies of an epidemiological, sociological, or educational nature.

There are a great many models in the HRQOL literature that have not been adequately tested or refined. Cross-comparisons across diseases could be done if authors had at least used a global HRQOL model as a starting point. In fact, many single-use models included the same concepts as the three global HRQOL models but labeled them differently. In the future, when a common global HRQOL model is not used, authors should clearly delineate why a context- or disease-specific model is preferred. Increasing the consistency in models used across studies would help increase our understanding of this important concept.

Of the 23 articles citing the three most common HRQOL models, most articles were descriptive, correlational, or literature reviews. Importantly, future HRQOL research should involve comparisons of intervention outcomes. Only one randomized controlled trial was found that used the most commonly cited Wilson and Cleary [12] model [25]. Although disease-specific or situation-specific models may be better for testing interventions, the global models should still be useful as a template and a jumping-off point for adaptations to specific contexts. In addition, using an existing model can advance the state of the science of HRQOL by contributing new information about the applicability of the selected model to research and practice, thus leading to model refinement such as the revised model proposed by Ferrans and colleagues [13]. This underscores the need to start with the best available HRQOL models and build upon them, rather than creating new models.

### Limitations

Our search strategies were limited to selected databases (PubMed, MEDLINE, CINAHL, and PsychINFO) and keywords (e.g., quality of life, health-related quality of life, conceptual framework, conceptual model, and theory). Given that standard keywords were used within each search engine, any article indexed by that search engine would have been captured; however, follow up manual searches and review of reference lists might have revealed additional citations. The search strategies were specifically designed to capture articles that were guided by or derived HRQOL models that were further analyzed in detail by the reviewers. All reviewers were doctorally-prepared and a research librarian assisted with the searches. Because the aim of our paper was to identify the most frequently-used HRQOL models found in the literature over the past 10 years, a complete synthesis of disease-specific models was not undertaken. Future work to analyze uniquely derived disease-specific HRQOL models may provide unique HRQOL domains that might further inform the three more commonly used HRQOL models. For example, Klassen et al. [18] qualitatively derived a HRQOL model for women who had undergone breast surgery. Their model consisted of six themes (satisfaction with breasts, satisfaction with process of care, satisfaction with overall outcome, psychosocial well-being, sexual well-being, and physical well-being). The satisfaction with process of care theme further informs both Ferrans and colleagues’ model [13] and the WHO ICF [3,20,21] as an important characteristic of the environment. Sexual well-being could further inform the functional status domains in all three models. Identifying domains that are unique to disease-specific models, or particular characteristics such as feedback or recursive patterns to address dynamic changes in HRQOL with time, may further inform or strengthen the rationale for using the three existing HRQOL models.

## Conclusion

In summary, based on this systematic review of the literature, Ferrans et al., [13] revision of Wilson and Cleary’s [12] model appears to have the greatest potential to guide HRQOL research and practice. We recommend Ferrans and colleagues’ [13] model because they added individual and environmental characteristics to the popular Wilson and Cleary [12] model to better explain HRQOL. Although the WHO ICF model has been considered a model of HRQOL, it is more of a mapping and classification framework than a guide for hypothesis generation in the area of HRQOL. Use of one model, such as Ferrans et al. [13] revised HRQOL model, will provide more opportunities for testing and refinement of the model and more evidence about which relationships among HRQOL concepts are common to different populations. Finally, and maybe most importantly, using one model will help in comparing HRQOL across studies and populations, contribute to the development of more intervention studies, and more quickly advance the science in the area of HRQOL.

## Abbreviations

HRQOL: Health-related Quality of Life; QOL: Quality of Life; WHO: World Health Organization; WHO ICF: World Health Organization International Classification of Functioning, Disability, and Health.

## Competing interests

The authors declare that they have no competing interests.

## Authors’ contributions

TB provided overall leadership and contributed to the conception and design, participated in the review and critique process, drafted sections of the manuscript, and revised it critically for intellectual content. SMM, JSC, JMB, JLO, KMH, MLE, &; JLW contributed to the conception and design, participated in the review and critique process, drafted sections of the manuscript, and revised it critically for intellectual content. KAH acquired articles for review, abstracted findings to tables, contributed to analysis and interpretation, provided reference management, and drafted sections of the manuscript. All authors read and approved the final manuscript.

## Supplementary Material

Additional file 1 Supplementary tables and references for the 100 Full-Text Articles reviewed for the manuscript entitled, “Systematic Review of Health-Related Quality of Life Models.” Click here for file
