# Supplementary material for: Systematic review of health-related quality of life models
Source: Health Qual Life Outcomes. 2012 Nov 16;10:134. doi: 10.1186/1477-7525-10-134 (PMC3548743; doi:10.1186/1477-7525-10-134)
Supplement: Additional file 1 — Supplementary tables and references for the 100 Full-Text Articles reviewed for the manuscript entitled, “Systematic Review of Health-Related Quality of Life Models.” [file 1477-7525-10-134-S1.docx]

**Table 2. Critique of Three Most Commonly Used HRQOL Models**

| **Criteria and Description (Bredow, [10])** | **Wilson & Cleary Model of HRQOL [17]** | **Ferrans et al. Revised Wilson and Cleary Model of**  **HRQOL [19]** | **World Health Organization International Classification of Functioning Disability and Health**  **(WHO ICF) [3]** |
| --- | --- | --- | --- |
| **Internal criticism** |  |  |  |
| **Adequacy**  Addresses a defined area   - Completeness - Gaps - Need for refinement | - Complete overall conceptualization of HRQOL from biomedical and social science perspectives - Gaps include management of therapeutic regimens and self-management - Refinement for specific practice situations needed. | - Expanded Wilson & Cleary’s model to better explicate individual and environmental factors - Gaps still include management of therapeutic regimens and self-management - Refinement for specific practice situations needed. | - Complete overall conceptualization of health from biomedical and social science perspectives - Gaps include determinants of health, management of risk factors, and self-management - Refinement for specific practice situations needed. |
| **Clarity**  Explicit components   - Concepts (components) defined - Explicit propositions   (Relationships)   - Explicit assumptions   (Beliefs) | - Main concepts well-defined, although individual and environmental characteristics not explained. - Explicit proposition that dominant relationships exist with the potential for reciprocal relationships. - Strength of the relationships of each component is unclear and with each additional relationship the complexity increases. - Other relationships were implied. - Explicit assumption that understanding relationships among these domains will lead to the design of optimally effective clinical interventions. | - Main concepts well-defined, including individual and environmental characteristics. - Explicit proposition that dominant relationships exist with the potential for reciprocal relationships. - Propositions were added with individual and environmental characteristics. - Nonmedical factors removed; described as part of individual and environmental characteristics. - Explicit assumption that understanding relationships among these domains will lead to the design of optimally effective clinical interventions. | - Main concepts well-defined, with the exception of overlap between activities and participation. - Explicit propositions exist with reciprocal relationships that can be used to map the constructs and domains. - Explicit assumption that model provides a multipurpose classification and can serve as a unified and standard language for health care workers, researchers, policy-makers, and the public. - Another explicit assumption is that model can be used to help plan interventions for functional goals and health, worldwide. |
| **Consistency**  Consistency   - Concepts   Congruency   - Assumptions (beliefs) - Propositions (relationships) | - Concepts consistently defined. - Assumptions were congruent - The figure depicts dominant directional relationships whereas the text mentions reciprocal and other non-depicted relationships. | - Concepts consistently defined. - Assumptions were congruent - Propositions were congruent. | - Concepts consistently defined. - Assumptions were congruent - Propositions were congruent. |
| **Logical development**  Based on previous work  Evidence supports | - Emerged based on research from biomedical and social sciences. - Relationships depicted don’t always hold true, research evidence supports lack of relationships in some instances (e.g., biological vs. symptoms) | - Revision of Wilson & Cleary - Emerged based on empirical evidence and the need for further clarity. | - Integration of medical and social models for a biopsychosocial approach. - Evolved over time from the WHO ICIDH model in 1980 to the WHO ICF in 2001, with the WHO ICF-CY for children and adolescents added in 2007. - Based on systematic field trials and international consultation. |
| **Level of development**  Level of abstraction (grand, middle range, or practice) | - Middle range but global | Middle range but global | Middle range but global |
| **External criticism** |  |  |  |
| **Complexity**   - Number of concepts - Parsimony - Complexity | - 5 main abstract concepts (biological/physiological, symptom status, functional status, general health, quality of life) - Parsimonious because used only 5 main concepts to explain abstract HRQOL. - Overall model is complex with multiple relationships | - 5 main abstract concepts with further development of the individual and environmental factors. - Parsimonious because used only 7 main concepts to explain abstract HRQOL. - Overall model is complex with multiple relationships | - 6 main abstract concepts (body functions, body structures, activity, participation, environmental factors, and personal factors). - Parsimonious because used only 6 main concepts to explain abstract health and health-related states. - Overall model is complex with multiple relationships |
| **Discrimination**  Unique theory of HRQOL with clear boundaries | - First HRQOL model to combine biomedical with social science - Unique to HRQOL - Boundaries are purposefully not clear as two theories are combined and the relationships between concepts are additive. - Hypotheses generation may help to clarify boundaries. | - Revised Wilson and Cleary’s HRQOL model - Unique to HRQOL - Clear boundaries and limited to HRQOL of individuals. | - Belongs to a family of WHO Classifications, with the WHO ICF being specific to functioning and disability. - Not unique to HRQOL. - Clear boundaries addressing health and health-related domains. - Does not cover non-health related circumstances. |
| **Reality convergence**   - Assumptions   “real world”   - Theory/model “makes sense” | - Moving from cellular level to quality of life in model seems more realistic than traditional biomedical model by itself. - “Makes sense” for real world application. - Assumptions are difficult to actualize | - Realism added with the incorporation of nonmedical factors into individual and environmental factors. - “Makes sense” for real world application. - Assumptions more realistic | - Assumptions seem true, realistic, and consistent. - “Makes sense” for real world application. |
| **Pragmatic**  Operationalized in real-life settings | Guided literature applied to real world settings:   - 3 literature reviews, - 4 descriptive, - 6 correlational, - 1 randomized trial, - 1 qualitative, - 1 mixed methods - 1 model revision (Ferrans)   Model testing in entirety rarely done   - Overall, generic and situation-specific measures exist - Response shift is a concern for general health and quality of life components | Guided literature applied to real world settings:   - 2 literature reviews - 1 instrument development   Model testing in entirety not done   - Overall, generic and situation-specific measures exist - Response shift is a concern for general health and quality of life components | Guided literature applied to real world settings:   - 3 literature reviews - 2 instrument development   Model testing in entirety not done   - Overall, generic and situation-specific measures exist - Response shift may also be a concern. |
| **Scope**   - breadth of theory/model - applies across ages (lifespan), health and disease conditions, cultures, socioeconomics, and individuals/families/ communities | - Broad model to explain complex nature of HRQOL - Could apply to individuals of all ages, life spans, health and disease conditions, and perhaps cultures depending on their orientation to the meaning of quality of life and general health. - May not apply to those who are unable to define their own general health or quality of life (e.g., infants, comatose), or those who have very limited functioning. - Primarily applies to individuals, less to families and communities. | - Further broadens Wilson and Cleary’s scope by expanding on individual and environmental factors | - Broad model to explain health and health-related domains for all people. - Could apply to individuals of all ages, life spans, health and disease conditions, and cultures across the world. - WHO ICF-CY specifically covers infants, children, and adolescents. - Focus is on individuals (with or without disabilities), families, communities, and populations. |
| **Significance**   - Potential impact on practice - Hypotheses lead to assessment or interventions | - Most widely cited HRQOL model - Guides HRQOL assessment toward a more comprehensive approach to improving HRQOL   Potential for intervention research but limited evidence exists to date.   - Because of the complexity of the model and lack of testing of the full model, supporting interventions would be difficult. | - Emerging citations for Revised HRQOL model - Guides HRQOL assessment toward a more comprehensive approach to improving HRQOL - Potential for intervention research but limited evidence exists to date. | - Emerging citations for the use of the WHO ICF for hypothesis testing (mainly instrument development). - As a clinical tool, can be used for needs assessments, matching treatments with conditions, and evaluating outcomes. - As a research tool, can be used for measuring quality of life, outcomes, environmental factors, or other constructs. - Potential for intervention research but limited evidence exists to date. More of a mapping and classification framework, rather than hypothesis generating. |
| **Utility**  Hypothesis generating for clinicians, researchers, epidemiologists, policymakers | Hypothesis generating for:   - Clinicians for a broader view of HRQOL than just biological factors and symptoms. - Researchers to guide measurement and intervention studies: - Potentially relevant to epidemiologists if using global measures across populations (e.g., SF-36). - More research evidence and emphasis on environmental factors needed to convince policymakers. | Hypothesis generating for:   - Clinicians for a broader view of HRQOL than just biological factors and symptoms. - Expands focus of article (audience) from physicians (Wilson & Cleary) to nurses and other health professionals (Ferrans). Model could be applied to any health care discipline. - Researchers to guide measurement and intervention studies. - Potentially relevant to epidemiologists if using global measures across populations (e.g., SF-36). - More research evidence and emphasis on environmental factors needed to convince policymakers. | Hypothesis generating for:  Clinicians for needs assessments, matching treatments with conditions, vocational assessment, and rehabilitation and outcome evaluation  Researchers to guide development of measures for outcomes, quality of life, or environmental factors  Epidemiologists to collect and record data for populations and management information systems  Policymakers to plan social security, compensation systems, and policies.  Educators to design curriculums that emphasize awareness and social action.  Although potential for hypothesis generation in these areas, there is currently limited evidence found in the HRQOL literature documenting these applications. |
